# Supplementary material for: Polyply; a python suite for facilitating simulations of macromolecules and nanomaterials
Source: Nat Commun. 2022 Jan 10;13:68. doi: 10.1038/s41467-021-27627-4 (PMC8748707; doi:10.1038/s41467-021-27627-4)
Supplement: Supplementary file 1 — Supplementary Information [file 41467_2021_27627_MOESM1_ESM.pdf]

Polyply, a python suite for facilitating simulations of (bio-) macromolecules and nanomaterials

Supplementary Information

Fabian Grünewald[1], Riccardo Alessandri[1,2], Peter C. Kroon[1], Luca Monticelli[3], Paulo C.T. Souza[3], Siewert J. Marrink[1]

[1] Groningen Biomolecular Sciences and Biotechnology Institute and Zernike Institute for Advanced Materials, University of Groningen, Groningen, The Netherlands

[2] Pritzker School of Molecular Engineering, University of Chicago, Chicago, IL 60637, USA

[3] Molecular Microbiology and Structural Biochemistry, UMR 5086 CNRS and University of Lyon, Lyon, France

Contents

Supplementary Note 1 2
Supplementary Note 2 3
Supplementary Note 3 3
Supplementary Note 4 4
4.1 Step 1-3: Template Generation & Estimating the volume from the radius of gyration 4
4.2 Step 4: Introducing restraints into the random walk-protocol 6
4.3 Step 5: Backmapping 8
Supplementary Note 5 10
5.1 Polymer melts at atomistic and CG level 10
5.2 DNA Virus additional Single-stranded DNA and circular single-stranded DNA 13
5.3 Polymeric lithium ion battery 14
5.4 Lipid vesicle with liquid-liquid phase separated interior 16
Supplementary References 17

Supplementary Note 1

The polyply library comprises a number of building blocks for polymers at different resolutions. Supplementary Table 1 gives an overview of all models implemented at the time of writing. An up-to-date list of libraries implemented in the program can be quarried with the following command:

```
$ polyply -list-lib
```

A list of monomers to a specific library can be obtained by running the command and specifying the name of the library as listed from the previous command:

```
$ polyply -list-blocks <name_of_library>
```

We note that while polyply has the ambition to offer a wide selection of monomers, which can be combined with each other, at the current stage not all monomers within a library are compatible with each other. Supplementary Table 1 gives an overview of the libraries and which combinations are allowed. When trying to generate a combination, which is not allowed, the program will issue a warning that the molecule consists of two disjoint parts. The GitHub wiki (https://github.com/marrink-lab/polyply_1.0/wiki) contains a list of monomer blocks that are being updated as monomers are added to the libraries. Furthermore it is highly recommended to check out the tutorials on the same page, which contain further important details on how to obtain appropriate itp files,for example how to add terminal groups for atomistic resolution polymers.

Supplementary Table 1. Overview of all polymers implemented in the polyply polymer library with corresponding abbreviations

Name	Abbreviation	Force-Field
Polyethylene glycol	PEO	OPLS-AA, 2016H66, Martini2, Martini3,
Polystyrene	PS	Martini2, Martini3, 2016H66
Polymethyl acrylate	PMA	Martini3, 2016H66
Polymethyl methacrylate	PMMA	Martini3, 2016H66
Polyethylene	PE	Martini3, 2016H66
Poly(3-hexylthiophene)	P3HT	Martini3, 2016H66, GROMOS53A6
Polyvinyl alcohol	PVA	Martini3, 2016H66
Dextran	DEX	Martini3
DNA nucleobases	DT, DG, DA, DC, DT5, DT3, DA5, DA3, DG5, DG3, DC5, DC3	Pambsc1
Amino Acids	3 letter code	Martini3

Supplementary Note 2

Supplementary Table 2 lists a number of scripts that facilitate the conversion of topology files from GROMACS to other MD programs. Such scripts are helpful if one wishes to perform simulations with the parameters generated by polyply with a software other than GROMACS. Note that OpenMM[1] can simply parse GROMACS topology files. Furthermore, InterMol[2] and Parmed[2] can also be used to convert other MD engine input files to GROMACS so that they can then be used with the structure generator.

Supplementary Table 2. List of utility scripts to perform GROMACS topology file conversions to input files of other MD engines.

Conversion	Program	Ref.
GROMACS→LAMMPS<br>GROMACS→Desmond	InterMol<br>https://github.com/shirtsgroup/InterMol	2
GROMACS→OpenMM	OpenMM can parse GROMACS topology files<br>https://github.com/openmm/openmm	1
GROMACS→CHARMM<br>GROMACS→AMBER<br>GROMACS→NAMD	Parmed<br>https://github. com/ParmEd/ParmEd	2

Supplementary Note 3

Polyply generates polymers from a residue graph. For a simple linear polymer that is simply the sequence, however, branched polymers or polymers which have a statistical distribution cannot simply be described by providing a linear sequence. Whereas for regular linear polymers polyply allows to provide the sequence directly as command line argument (-seq) for the gen_params tool, more complex residue graphs can be provided in the form of a networkx graph json-file. To further facilitate generating parameters for these more complex polymer topologies we have implemented the 'polyply gen_seq' auxiliary tool. The gen_seq tool can be used to create and manipulate residue graphs from the command line interface. More details on the syntax and usage of the sequence generation tool can be found on the GitHub wiki page (https://github.com/marrink-lab/polyply_1.0/wiki/Synatx:-gen_seq) or the help menu by running `polyply gen_seq -h`.

Supplementary Note 4

This section offers additional details behind the algorithms used in the structure generation module of the code.

4.1 Step 1-3: Template generation & volume estimation from the radius of gyration

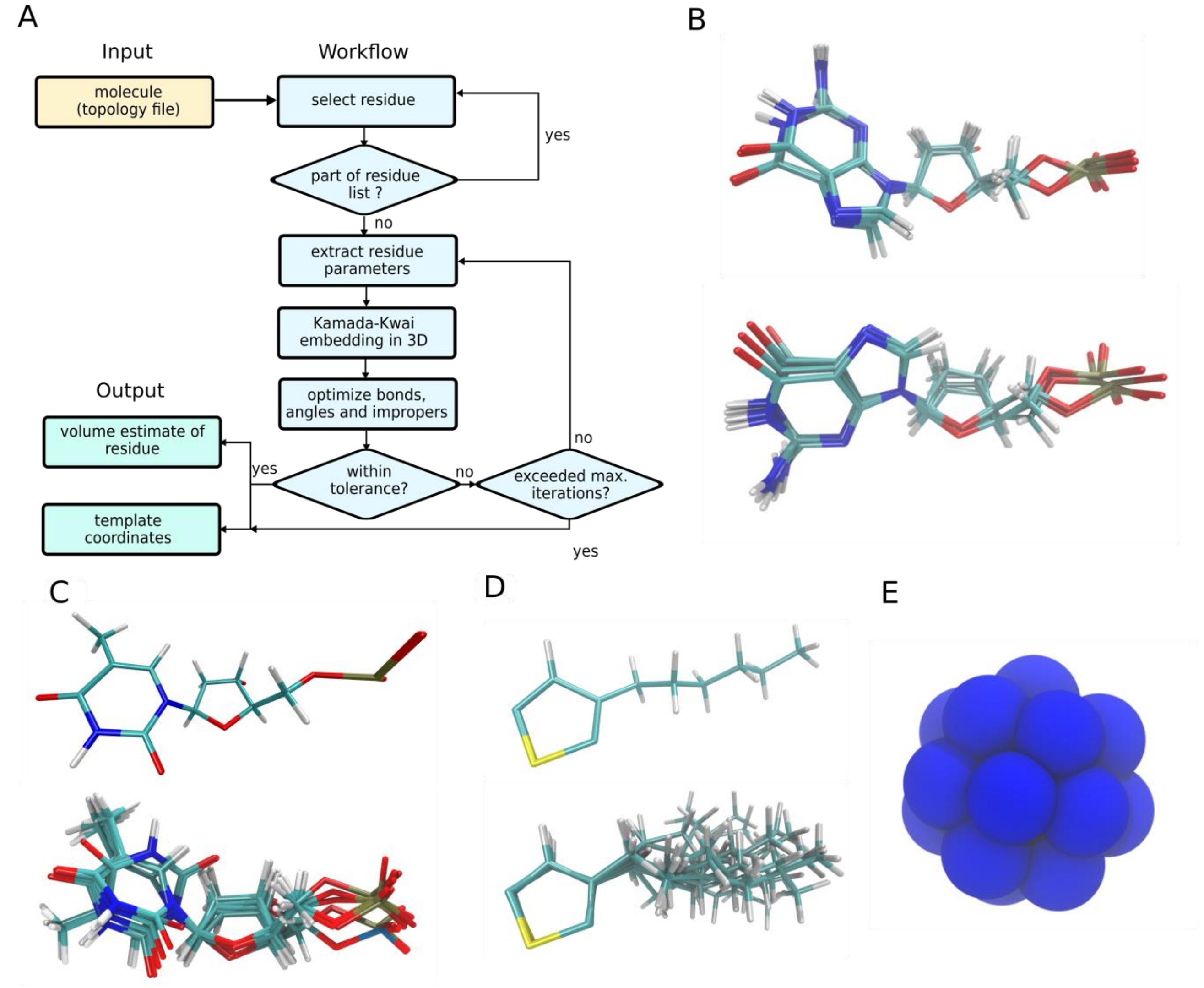

Supplementary Figure 1. Template Coordinate Generation; A) general workflow for template generation; B) all-atom coordinates generated by polyply for Deoxyguanosine showing two sets of conformers distinguished by rotation of the Guanine group with respect to the bond to the deoxyribose; C) all-atom coordinates generated by polyply for Deoxyadenosine; top shows a single conformation and bottom shows clustering of 10 conformers D) all-atom coordinates for Poly(3-hexylthiophene-2,5-diyl)[3]; top shows a single conformation and bottom shows clustering of 10 conformers; E) Martini CG C_{60} molecule[4] generated by polyply

In polyply, the volume of a residue is directly computed from the residue coordinates and non-bonded interactions. Before the volume can be estimated, residue coordinates for each unique residue in the system are generated by embedding the graph description into 3D coordinates using a two-step optimization

procedure. The detailed procedure is outlined in Supplementary Figure 2A. After extracting all bonded interactions belonging to the residue from the topology file, an initial set of coordinates is generated by a Kamada-Kawai embedding. This form of embedding only uses the molecular graph information but is superior to random coordinates as it places bonded atoms/beads in close proximity and is even able to keep rings connected and close to within a plane. Using these coordinates, the geometry is optimized by minimizing the following objective function:

$$\sum_{\substack{bonds \\ constraints}} 10^4 \times (d - d_{ref}) + \sum_{angles}(\theta - \theta_{ref}) + \sum_{\substack{improper \\ dihedrals}}(\varphi - \varphi_{ref}) \quad (1)$$

In principle the algorithm optimizes bonds, angles, constraints and improper dihedral angles such that the value in the molecule matches the equilibrium value. However, as typical molecules have many more angles and dihedrals than bonds, we found it advantageous to scale the contribution of the bonds by an empirical factor of 10^4. Note that the algorithm will also correctly take into account and place GROMACS virtual sites. An optimization within polyply is only accepted if every single bond or constraint is within 0.5Å of the equilibrium value and every angle is within 5 degrees of the equilibrium value otherwise a warning is issued. Proper dihedral angles are not optimized as they are typically flexible and conformers will easily relax to the desired conformation.

We have benchmarked the coordinate generation protocol by generating 20 template residues for each of the blocks in the Parmbsc1[5], GROMOS 2016H66[6] and GROMOS 53A6[3,7] force-field cumulating to 460 different templates at the all-atom and united atom level. None of the optimizations failed, meaning that the geometries are reproduced for all templates within the tolerances outlined previously. Supplementary Figure 1 B-E shows some typical geometries obtained from the template algorithm. For Deoxyguanosine (Supplementary Figure 2B) we have aligned 10 conformers to show the flexibility of structures obtained from the algorithm. For the case of Deoxyguanosine typically two different conformers are obtained that differ from each other by rotation of the guanine group around the bond to the ribose fragment. Both are valid starting points for an MD simulation. For Deoxyadenosine (Supplementary Figure 2C) and P3HT (Supplementary Figure 2D) the top panels show a single typical conformer and the lower panels RMSD aligned conformers as generated by polyply. We clearly see that the alkyl chain in P3HT has a larger amount of conformers given the larger rotational flexibility. For both molecules the ring fragments are clearly resolved and reproduced. Finally, as shown in Supplementary Figure 1E polyply can also generate coordinates for simple nanoparticles. At the Martini CG level C_{60}[4] is optimized without issues. Overall we conclude that the residue templates are built to high accuracy in a reliable manner using this two-step algorithm. This makes it possible to estimate a realistic volume for each residue while at the same time avoiding unrealistic conformations and high energy states at the residue level.

After the residue coordinates are obtained the volume is estimated from the radius of gyration of the residue, however, taking into account the van-der-Waals radius of the atom/bead. The formula for the radius of the volume becomes:

$$r_{res} = \sqrt{\frac{1}{N}\sum_k (1 + r_{vdW}) * (r_k - r_{COG})} \quad (2)$$

Essentially we compute the radius of gyration but add a van-der-Waals contribution in the direction of the position vector from the center of geometry (COG). This makes sure that we capture some volumetric component in the radius of gyration. This procedure was selected for two reasons: 1) the radius of gyration is essentially a moment of inertia neglecting the mass. Thus it captures a radius that the residue has when rotating about the center of geometry. In other words, it could be described as a rotational volume as opposed to the real volume of the residue. When generating an initial starting structure, we need to make sure that there is no direct overlap between any atoms when transforming from the super CG model to target resolution. However, using the actual volume as for example computed from a voxelized approach that sums the volume of the intersecting vdW-spheres, we frequently find overlaps. 2) The above description can take into account larger ligands, conjugated systems, and residues which are much larger than a typical polymer radius. Imagine computing the volume for C_{60}, which is completely round but hollow inside. If we were to compute the volume using a voxelized approach by summing the vdW radii we would greatly underestimate the volume. The same holds for example for a lipid that is treated as one residue or an extended conjugated system. Ultimately, it is a practical choice where we observe that our radius of gyration approach works well.

4.2 Step 4: Introducing restraints into the random walk-protocol

A self-excluding random-walk typically is not able to reproduce specific conformations like ring-closures or very straight paths. This can be a problem when trying to generate coordinates for macromolecules. For example, molecules with high persistence length require comparatively straight paths. Within polyply we have augmented the random-walk protocol with a restraint algorithm which is able to effectively force specific distance restraints between residues of the system, while keeping the rest of the walk random. This allows us to also implement a protocol for taking into account the persistence length of polymers. In this section we will outline the algorithm behind that implementation as well as some initial benchmarking results.

Let us consider the example in Supplementary Figure 2A. Given that the nodes one (reference node) and six (target node) shall be restraint at a distance r (Supplementary Figure 2A) the algorithm computes an upper and lower bound for each node on the path from one to six (i.e. 2,3,4,6). This upper and lower bound are specified as the maximum and minimum distance each of the nodes can have, measured in relation to the position of node one. The upper bound is computed as the graph distance of each node to the reference node multiplied by the average step length of the random-walk plus the distance r (Supplementary Equation 3).

$$upper_{bound}(node) = r_{graph}(ref; node) \times \overline{step} + r_{dist} \quad (3)$$

The lower bound is computed as the average step length that is needed to connect node one to node 6 at a distance r multiplied by the graph distance of that node to the reference node.

$$lower_{bound}(node) = \frac{r_{dist}}{r_{graph}(ref;\, target)} * r_{graph}(ref;\, node) \quad (4)$$

So for the example the algorithm would implement 4 upper and lower bounds for the nodes 2-6. To help convergence, a tolerance may be added to each of the bounds by the user. Note that given the two constraints, the algorithm will make sure that the two nodes are always at a distance r ± one step length of the random-walk.

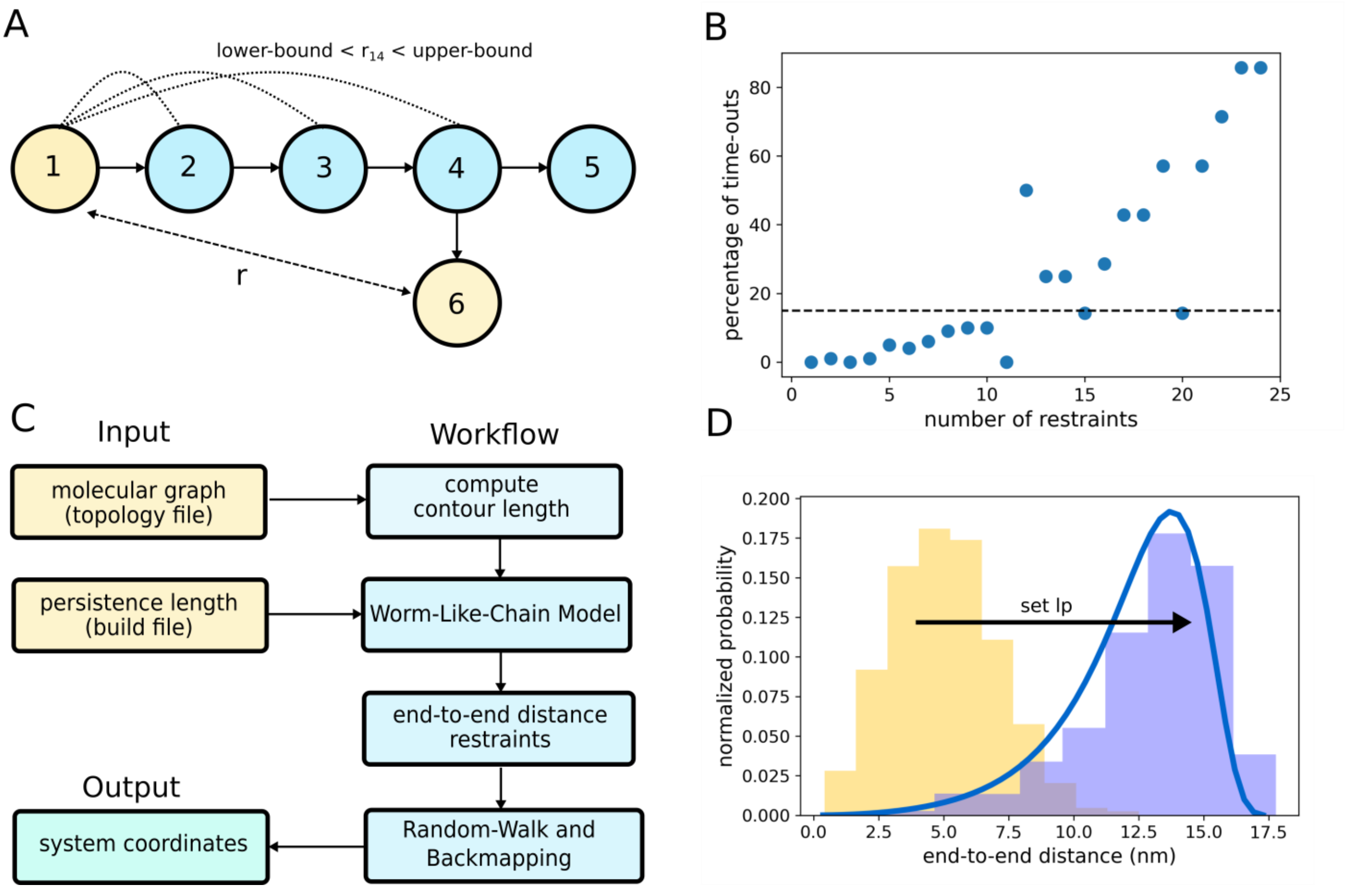

Supplementary Figure 2. Distance restraints. A) Concept of distance restraints in polyply. To achieve node 1 and 6 to be at distance r each node on the way has an upper and lower distance it can assume in relation to node 1. Those bounds are computed from the graph distances and average step length according to equation 3 and 4. B) Benchmarking of restraint algorithm, for a given number of restraints it shows the percentage of times the algorithm took longer than 5 min to find a solution for a chain of 100 residues. C) Workflow behind the end-to-end distance sampling algorithm for rigid polymers within polyply D) End-to-end distances of P3HT melts generated without setting the persistence length (gold) and setting the persistence length (blue). The thick curve corresponds to the expected end-to-end distance from the WCM.

Supplementary Figure 2B shows some benchmarking results on the algorithm. To benchmark the algorithm we have taken a linear chain of 100 residues and applied between 1 to 25 distance restraints and generated coordinates. The distance restraints are taken from a melt of 100 chains also produced by polyply to ensure that the distances are realistic (i.e. can be reached within a random-walk). Subsequently for each batch we generated 100 random replicas of distance restraints. If the algorithm was able to satisfy these restraints in less than 5min, we considered it a success and otherwise the program was terminated.

Supplementary Figure 2B shows the percentage of these time-outs as function of the number of restraints. We can see that the algorithm is able to very efficiently generate conformations for up to 5 distance restraints with less than 10% failure rate. This means we are able to very effectively force for example end-to-end distances.

The more distances are applied on the molecule the more time-outs are registered. With up to about 15 restraints the fail rate is less than 15%. With more than 15 distance restraints the algorithm becomes progressively less effective. However, one should note that this does not mean that it doesn't converge eventually. It simply means it cannot converge within the 5min time limit. Considering that the algorithm is still able to find solutions at all within 5min for cases, where a quarter of all residues are fixed at a specific distance we find very encouraging. In addition, there is room for future improvement by for example optimizing the number of rewind steps or the tolerance.

Given that the algorithm can very reliably produce one distance, it was now possible to implement specific end-to-end distances for polymers with high persistence lengths and even generate macrocycles. Supplementary Figure 2C shows the general workflow for taking into account the persistence length of a molecule. As input we only require the persistence length and the molecular graph, which is extracted from the topology file. Subsequently, the contour length of the generic CG model is computed. From the persistence length and contour length, we compute the probability distribution function of end-to-end distances given the worm-like-chain model. In future, other models could be implemented as well. Subsequently, the distribution is sub-sampled to generate an end-to-end distance for each polymer for which coordinates shall be generated. Coordinates are then generated via the RW protocol taking the end-to-end distance into account followed by backmapping to get the coordinates at target resolution.

Supplementary Figure 2D shows the example of a P3HT melt. The gold distribution shows the end-to-end distances for P3HT with 50 residues in the melt from polyply. However, it is known that P3HT has a high persistence length (~3nm in solution). According to the WCM model, the end-to-end distances should be larger as indicated by the blue curve. Using the persistence algorithm within polyply the blue distribution is generated by setting a persistence length of 3nm. It is interesting to note that already the golden distribution is significantly shifted from the flexible polymers of similar length, meaning that the generic CG model already captures some rigidity. However, by using the restraint algorithm a clearly superior result is achieved.

4.3 Step 5: Backmapping

The backmapping procedure within polyply is a template based approach as outlined in the main paper. Supplementary Figure 3A gives the detailed stepwise workflow. We note a few things: To avoid high energy and artificial structures such as for example interlocking rings, the templates are scaled after rotational alignment. Therefore, the structure that is generated by polyply contains all coordinates but shrunk towards the center of each residue. During the energy minimization step, which the user has to conduct, the coordinates expand. This typically allows residues which would be in rather close contact when placed 1:1 to actually move out of the way. This procedure has been previously very successfully used in the backwards backmapping program.[8] The success of this method is further demonstrated by the fact that for

all systems we could conduct an energy minimization without any artificial Hamiltonian scaling or special adjustments.

Nevertheless, to further support this procedure we analyzed the PS melts generated in example 1. We find that the closest distance between two rings in the PS belongs to a sandwich stack motive of two directly neighboring residues with a distance of about 3.2Å (Supplementary Figure 3B). As a reference, we then also constructed a motive with two interlocking Benzene rings (Supplementary Figure 3C). Here we find the center of mass (COM) distance to be about 2Å.

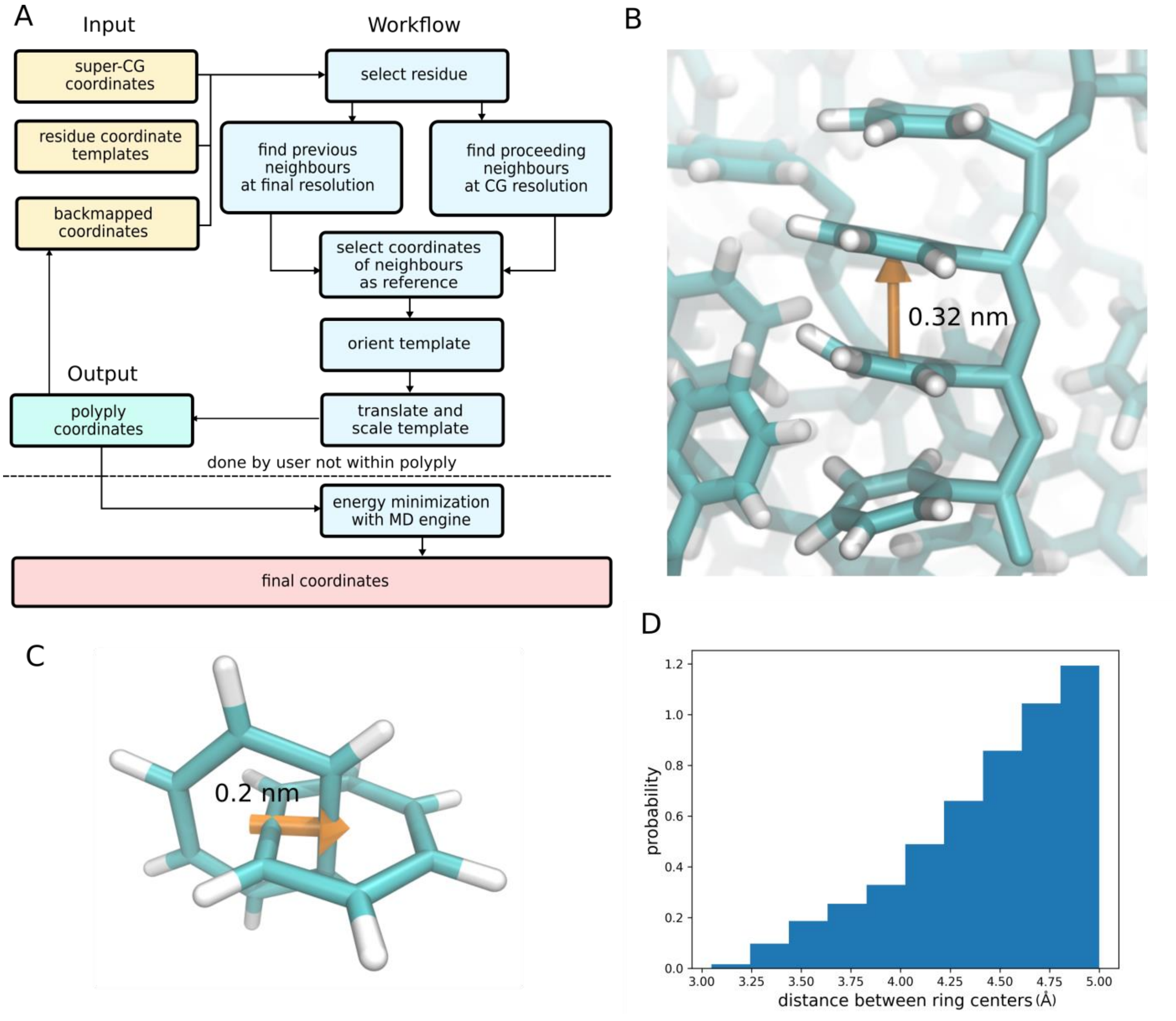

Supplementary Figure 3. Backmapping within polyply. A) Workflow of the backmapping protocol within polyply; B) Minimum stacking distance measured from COM observed in polyply generated PS; C) Minimum stacking distance from COM for two interlocking rings; D) COM distance distribution between rings from polyply PS melts with 10000 residues.

Analyzing the melt simulations for PS we find that no melt produces structures where the COM of the rings are closer than 3Å showing that no interlocking rings are produced. Likewise, visual inspection of the generated DNA molecules constructed leads to the same conclusion. Thus we are confident that the algorithm will produce good low energy starting structures for MD simulations.

Supplementary Note 5

5.1 Polymer melts at atomistic and CG level

End-to-end distances were computed from two models: the worm-like chain model (WCM) and the hindered rotation model (HRM). In case of the WCM model the distribution was computed as suggested previously by Lee et al. and subsequently the expectation value of the distribution was calculated to obtain the average end-to-end distance.[9] In the case of the HRM the mean square end-to-end distance is given by equation 2.[10]

$$< R^2 >= n * l^2 * C_\infty \quad (5)$$

In Supplementary Equation 5 C_∞ is Flory's characteristic ratio, which can be measured experimentally or computed from the persistence length. Furthermore, n is the number of bonds with length l. The distribution of the WCM model is computed from the persistence length (l_p), which was obtained from Flory's characteristic ratio (c.f. eq2) and the contour length, which was computed from the bond length (l) and angle (θ) of the atomistic model parameters (c.f Supplementary Equation 6 and 7).

$$l_p = \frac{C_\infty}{2*l} \quad (6)$$

$$R_{max} = n \times l \times cos\frac{\theta}{2} \quad (7)$$

Sometimes, for the same polymers several different values for the persistence lengths and characteristic ratios are reported in literature. Whereas for polymers such as PS the values of the characteristic ratio range from 8.24Å[11]-9.6Å[12], which is rather close together, for others such as PEG or PVA quite different values are reported. For PEG the values range between 4.2Å and 11.2Å.[13] However, there appears to be an agreement between simulations and theory[11,13–15] for a lower value between 2Å-6Å. These values fit better with the persistence lengths and end-to-end distances obtained from simulation and theoretical calculations. Therefore, we have taken the average of three theoretical calculations[13] as reference value. Similarly, for PVA estimates range from 3.4Å-8.5Å[11,16]. In this case it is unclear which value is more reliable. We have chosen the value of 4.81Å as recently reported for CHARMM all-atom simulations[11], as that was recorded for the melt. Bond lengths and angles were taken from the GROMOS 2016H66 model, for which the carbon-carbon bond length is 1.53Å and the angle theta is 70 degrees. For PEG the average of the C-O and C-C bond was taken as is custom. Supplementary Table 3 collects the values for the characteristic ratio, target density together with the corresponding temperature as well as bond length and theta angles.

As outlined in the main text, we find a good match between the average end-to-end distances generated by polyply and the two models presented previously. For the WCM, however, we not only have infor-

mation about the predicted average values but also the distribution associated with that model. Supplementary Figure 4 compares the distributions obtained by the WCM model and polyply for PEO using the GROMOS force-field (panel A) and Martini force-field (panel B) for all four chain lengths studied. We have chosen to use PEO as an example, because it has been previously reported that PEO is well described by the WCM model.[9]

Supplementary Table 3. Physical constants of polymers used in the end-to-end distance calculation. Densities at 413K are taken from ref 10 *and densities at room temperature are the amorphous densities taken from ref*[17]*.* C_∞ *values are averaged over several references unless stated otherwise. *average of theoretical values reported in ref* 13.

polymer	C_∞ (Å)	T (K)	ρ (g cm^{-3})	θ degree	l_b (Å)
PS	9.11[10,11,18]	413	0.969	70	1.53
PE	7.34[10,11]	413	0.784	70	1.53
PMA	8.15[11,19]	298.15	1.100	70	1.53
PEO	4.5*	413	1.064	70	1.48
PMMA	8.2[10]	298.15	1.100	70	1.53
PVA	4.81[11]	298.15	1.200	70	1.53

Overall the agreement is good, but we note that the distributions of the AA model are slightly shifted for the longer chain lengths. This is a common trend we see, however, it should also be noted that in comparison to the WCM model the higher molecular weights are worse, whereas in comparison to the generally more accurate HRM model the average end-to-end distances are in better agreement.

For each of the GROMOS 50 repeat unit melt chain systems, we ran a 10ns MD simulation under constant pressure. Supplementary Figure 5A shows the densities and Supplementary Figure 5B the end-to-end distances as function of simulation time. The temperature for all simulations was 513K to ensure that all simulations are well above the melting point of the polymer species. For each of the MARTINI 50 repeat unit systems we ran 50ns of MD simulations. The corresponding results are shown in the lower two panels. As apparent from the left panels the end-to-end distance quickly relaxes to the force-field native average end-to-end distance and then fluctuates around the average. However, no strong relaxations can be observed suggesting the end-to-end distances from polyply are a good starting point. Similarly, as shown in right panels, the densities take typically less than few ns to relax to their target values at the temperature of interest.

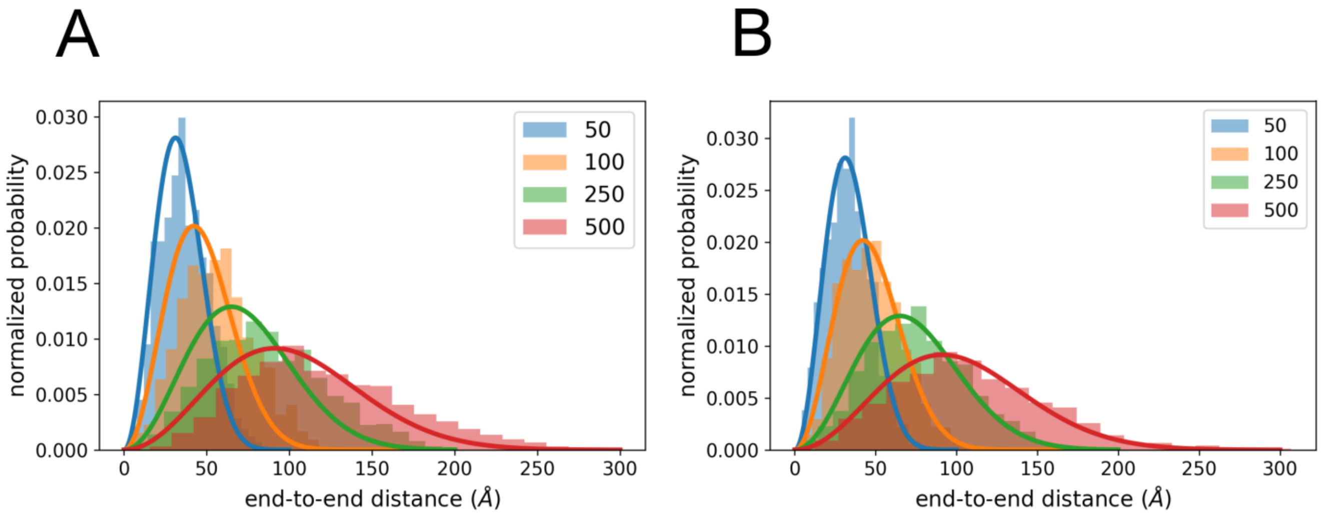

Supplementary Figure 4. End-to-end distance distribution of PEO obtained from the WCM (solid curves) and polyply (histogram) for 4 chain lengths. Panel A shows the GROMOS based distributions and panel B those obtained for Martini.

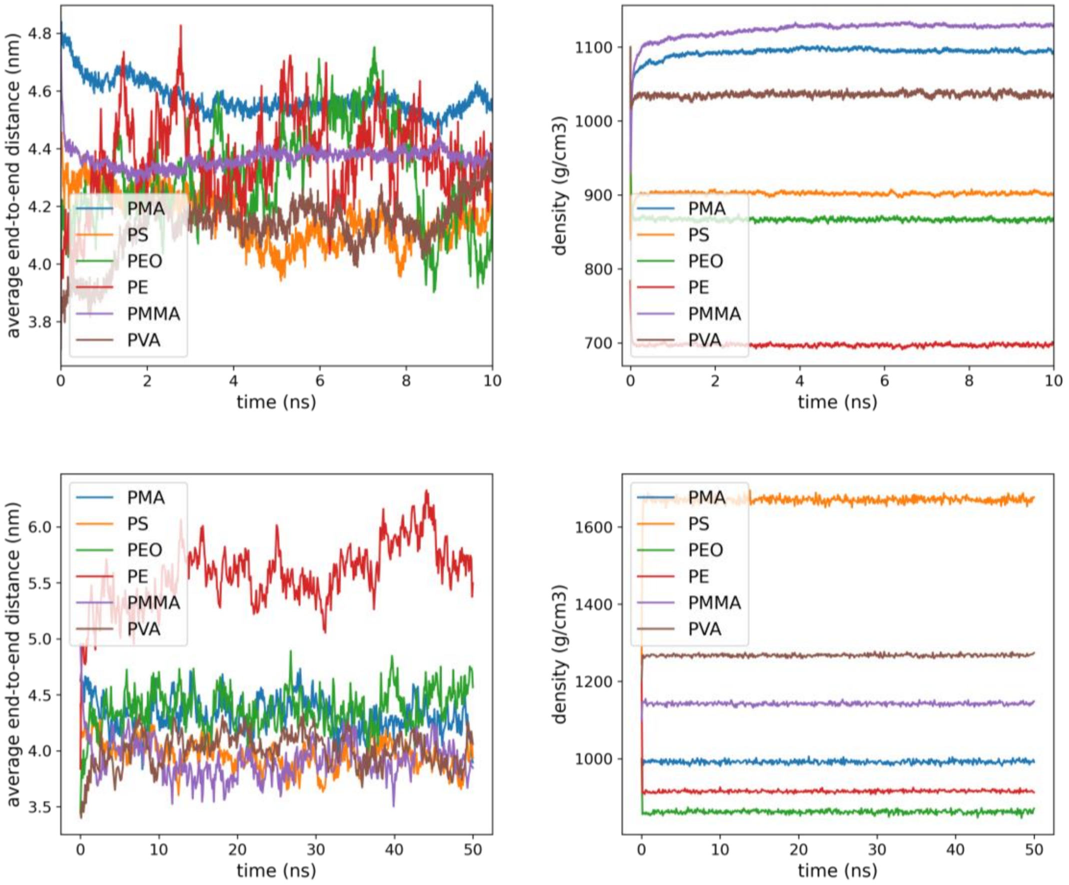

Supplementary Figure 5. Densities and end-to-end distances for equilibration runs of melt systems with 50 residues each at 513K. Top two panels are for GROMOS, bottom two panels for Martini.

5.2 DNA test cases

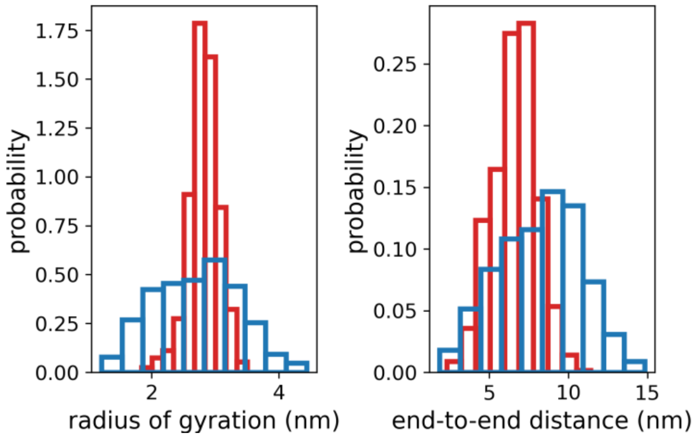

Supplementary Figure 6. Distributions of the radius of gyration and end-to-end distance from all-atom simulation (red) and polyply generated structures (blue).

Supplementary Figure 6 shows the distributions of the radius of gyration and end-to-end distance of an AA simulation of 32 bases polyT in comparison to 200 structures generated with polyply. Whereas we see that the polyply distributions are somewhat wide, there is very good overlap between the two sets suggesting the polyply conformations are a good starting point. Furthermore, we note that the AA force-field predicts a radius of gyration (2.8±0.5nm) lower than experimentally expected (3.8±0.1nm)[20]. Thus we used a lower persistence length (1.4nm) to generate the polyply conformations.

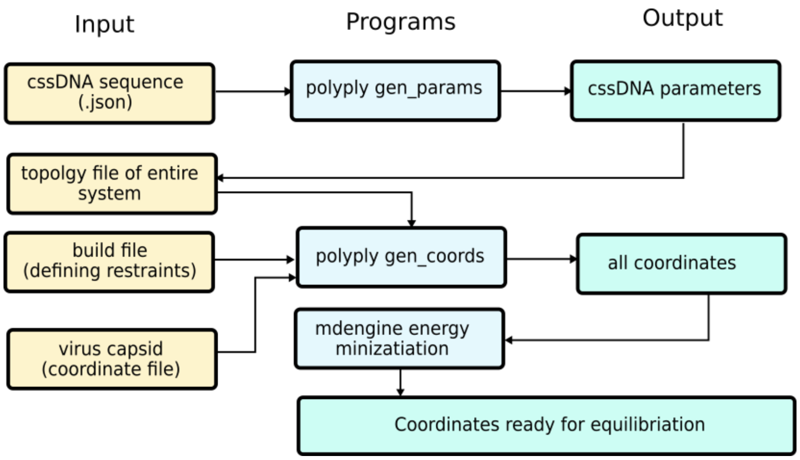

Supplementary Figure 7. Workflow for generating porcine circular ssDNA inside virus capsid.

As a further complex test case, we have generated the circular genome of the porcine virus at the all-atom level. Supplementary Figure 7 shows the general workflow for this example. First, the sequence was obtained from the database as well as the crystal structure of the capsid. Subsequently, the input parameters for the DNA are generated from the sequence with the gen_params tool using the PamSC1 force-field. Parameters for the protein crystal structure are taken from Amber14[21] and are generated with gmx pdb2gmx from the GROMACS[22] tools and then included as part of the system topology. In the next step,

coordinates are generated by using the protein capsid as well as a spherical geometry restraint as boundaries for the DNA. While the protein capsid is a natural boundary for the DNA the spherical restraint simply makes the algorithm a little faster compared to having to evaluate every overlap with the capsid.

For each nucleobase placed, a sodium ion is placed close to the nucleobase using the ligation feature within polyply. Placing neutralizing sodium ions is essential to keep the initial structure stable. To our knowledge the salt concentration inside the capsid is unknown, so a neutralizing concertation was used. The capsid itself also contains charged residues, we have added a neutralizing amount of chlorine ions randomly in the box to achieve a completely charge neutral box. Finally, the cycle option is used to tell polyply to make a circular molecule DNA molecule. We have not set an persistence length on the circular DNA because the persistence length of DNA depends on the salt concentration and at very high salt concentration long DNA is observed to even form flexible coil like conformations.[23] A rough back of the envelope calculation assuming a spherical virus of 7.5nm radius yields a salt concentration of about 2 mol/L inside the virus. This would be a relatively high concentration where we consider a random coil starting structure justified.

Once the circular DNA, sodium and chlorine ions are generated, gmx solvate is used to add water to the system. Polyply is very efficient at building macromolecules but less efficient at placing solvents. Therefore we used gmx solvate. Subsequently the system was equilibrated in three steps first using a low time-step (0.1fs) and flexible bonds, followed by a1fs time-step run with constraint bonds. For both equilibrations the SD integrator was used. Finally, the production run was done at 2fs using constraints on all bonds and the normal leap-frog integrator. Within the 60ns production run the system was stable and we see an exchange of ions between the inside of the capsid and the outside suggesting that there is an optimal salt concentration for the inside of the capsid.

5.3 Polymeric lithium ion battery

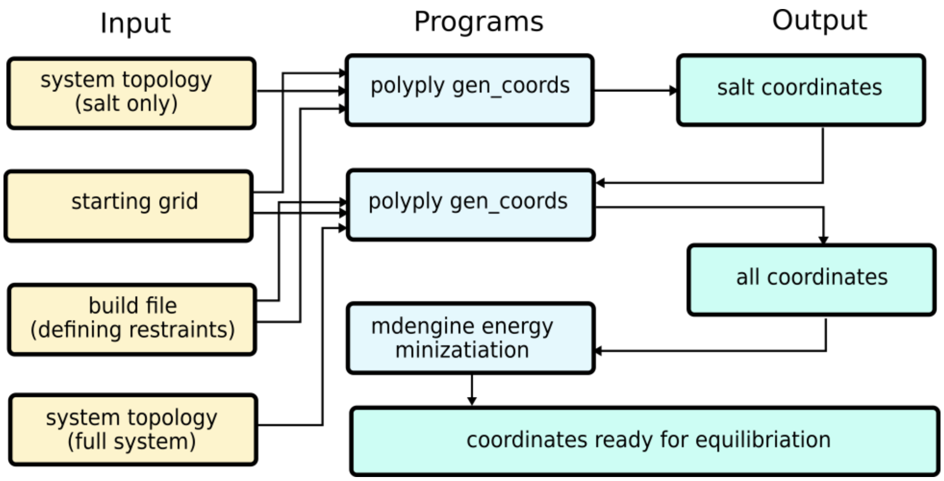

Supplementary Figure 8. Workflow for generating PS-b-PEO LiTFIS doped battery at the Martini2 level resolution.

After setting up the system with polyply we further characterized the microphase-separated block-copolymer by computing the density profiles of the components along the z-axis. The result is shown in

Supplementary Figure 9. It clearly shows distinct alternating domains of PEO and PS with some degree of mixing at the interface. From the lower panel we also see that the salt is enriched inside the PEO domain relative to the edges of the PEO domain. This form of salt channel formation has been reported previously in simulation[24] and has been proposed as one possible explanation for experimental conductance trends in PEO-b-PS based batteries.[25] Furthermore the domain spacing can be computed from the total box size. After equilbration the total box dimension in z is stable with an average value of 63nm. Considering we see three domains the average domain spacing is about 21nm in good agreement with the reported experimental value of 20nm for this systems.[26]

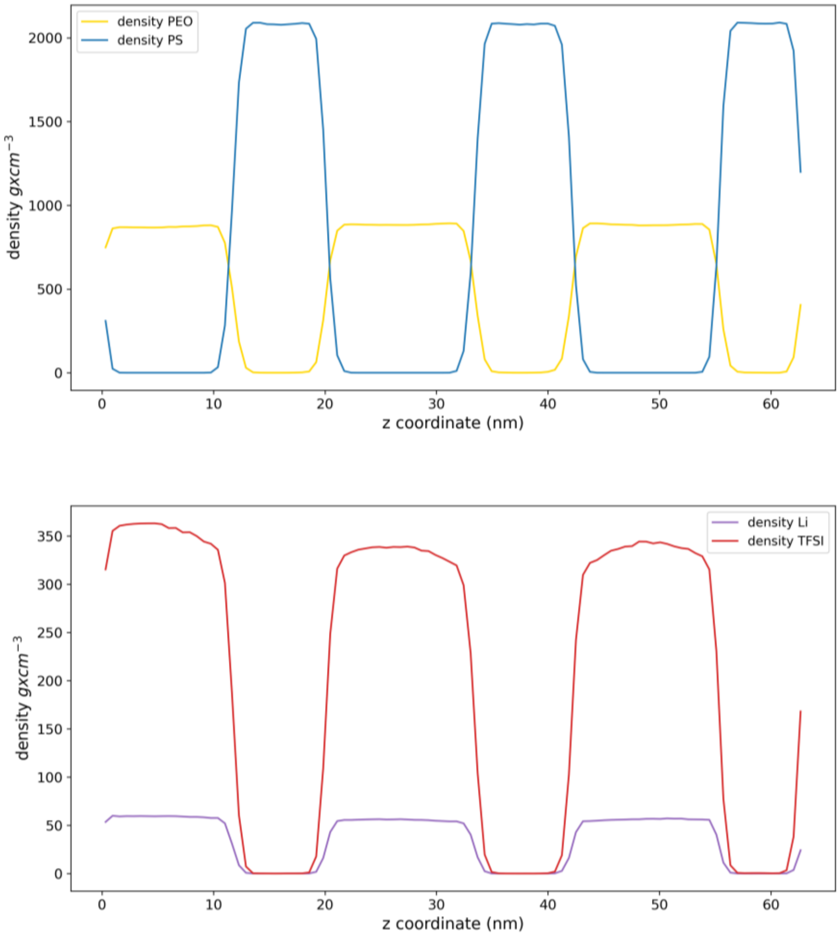

Supplementary *Figure 9. Density profiles of PS-b-PEO LiTFSI doped battery after equilibration of 50ns.*

5.4 Lipid vesicle with liquid-liquid phase separated interior

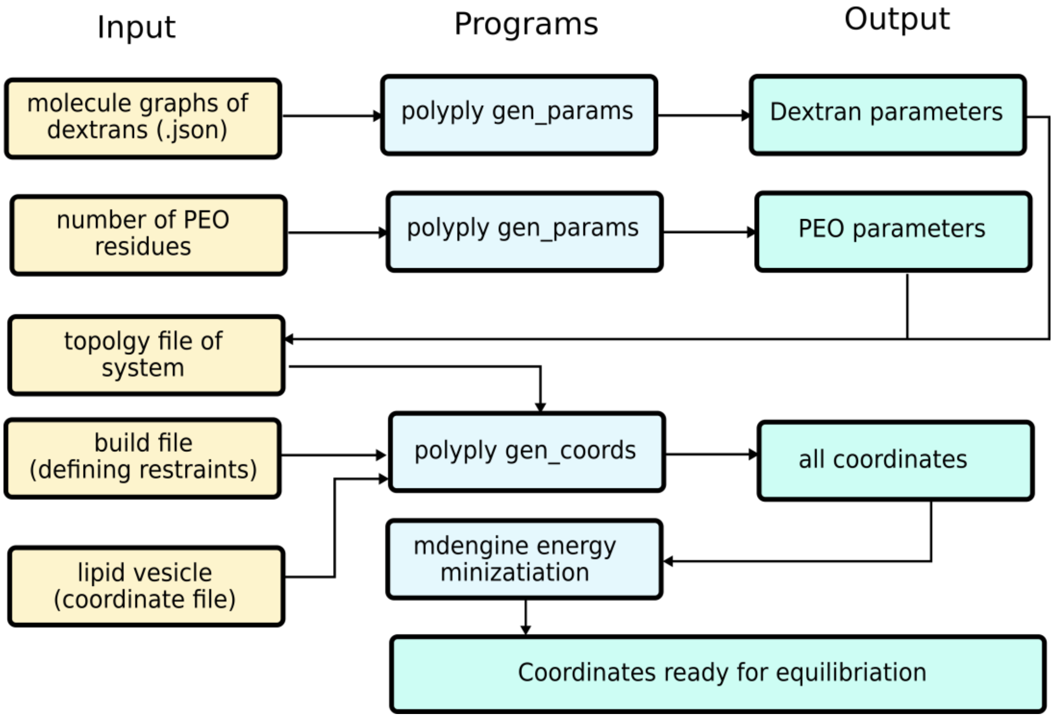

Supplementary Figure 10. Workflow for generating a vesicle with liquid-liquid phase separated Dextran PEO.

Dextran is in general a branched and polydisperse polysaccharide. The degree of branching is reasonably well characterized with literature agreeing that about 5% linkages should be present for molecular weights lower than 100,000 g/mol.[27,28] Unfortunately the polydispersity is less well characterized and depends on the synthesis method and if the polymer was fractionated afterwards. As we are only interested in the polydispersity for the sake of demonstrating polyply's capability of dealing with polydisperse polymers, we modeled the molecular weight distribution as follows: We assume that the synthesis follows the reaction kinetics of linear condensation polymers. For this type of reaction the molecular weight distribution can be computed only from knowing the extent of reaction (p in Supplementary Equation 8).[10]

$$prop(N,p) = N \times p^{N-1}(1-p)^2 \quad (8)$$

Subsequently, we change the extent of reaction such that the number average molecular weight becomes about 65, which is the weight used in the experimental system we aim to model.[29] The obtained polydispersity index in that case is about 1.5 which compares reasonably well to the value of 1.8 found in literature[30]. Supplementary Figure 11 shows the distribution and a histogram of the 500 samples drawn. Note that the smallest allowed polymer was 2 residues.

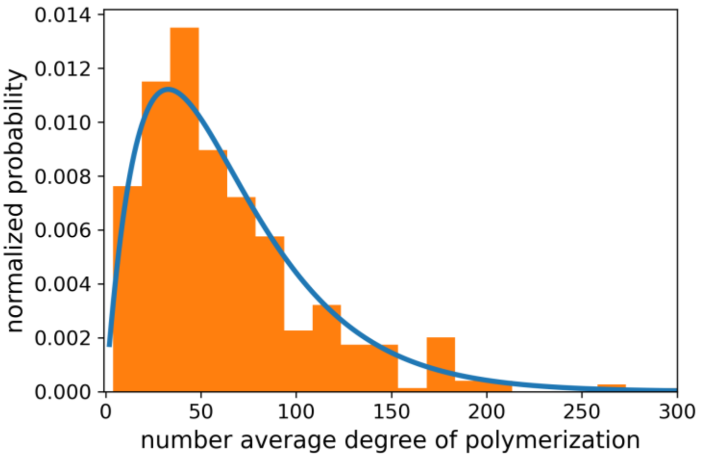

Supplementary Figure 11. Molecular weight distribution of dextran from equation 8 (solid line) and the samples drawn for structure generation.

Supplementary References

1. Eastman, P. *et al.* OpenMM 7: Rapid development of high performance algorithms for molecular dynamics. *PLOS Comput. Biol.* **13**, e1005659 (2017).

2. Shirts, M. R. *et al.* Lessons learned from comparing molecular dynamics engines on the SAMPL5 dataset. *J. Comput. Aided. Mol. Des.* **31**, 147–161 (2017).

3. Alessandri, R., Uusitalo, J. J., De Vries, A. H., Havenith, R. W. A. & Marrink, S. J. Bulk Heterojunction Morphologies with Atomistic Resolution from Coarse-Grain Solvent Evaporation Simulations. *J. Am. Chem. Soc.* **139**, 3697–3705 (2017).

4. Monticelli, L. On Atomistic and Coarse-Grained Models for C $_{60}$ Fullerene. *J. Chem. Theory Comput.* **8**, 1370–1378 (2012).

5. Ivani, I. *et al.* Parmbsc1: A refined force field for DNA simulations. *Nat. Methods* **13**, 55–58 (2015).

6. Horta, B. A. C. *et al.* A GROMOS-Compatible Force Field for Small Organic Molecules in the Condensed Phase: The 2016H66 Parameter Set. *J. Chem. Theory Comput.* **12**, 3825–3850 (2016).

7. Oostenbrink, C., Villa, A., Mark, A. E. & Van Gunsteren, W. F. A biomolecular force field based on the free enthalpy of hydration and solvation: The GROMOS force-field parameter sets 53A5 and 53A6. *J. Comput. Chem.* **25**, 1656–1676 (2004).

8. Wassenaar, T. A., Pluhackova, K., Böckmann, R. A., Marrink, S. J. & Tieleman, D. P. Going backward: A flexible geometric approach to reverse transformation from coarse grained to atomistic models. *J. Chem. Theory Comput.* **10**, 676–690 (2014).

9. Lee, H., de Vries, A. H., Marrink, S.-J. & Pastor, R. W. A Coarse-Grained Model for Polyethylene Oxide and Polyethylene Glycol: Conformation and Hydrodynamics. *J. Phys. Chem. B* **113**, 13186–13194 (2009).

10. Colby, R. H. & Rubinstein, M. *Polymer physics*. (Oxford University Press, 2003).

11. Choi, Y. K. *et al.* CHARMM-GUI Polymer Builder for Modeling and Simulation of Synthetic Polymers. *J. Chem. Theory Comput.* **17**, 2431–2443 (2021).

12. Mays, J., Hadjichristidis, N. & Fetters, L. J. Characteristic Ratios of Model Polydienes and Polyolefins. *Macromolecules* **17**, 2723–2728 (1984).

13. Kugler, J., Fischer, E. W., Peuscher, M. & Eisenbach, C. D. Small Angle Neutron Scattering Studies of Poly (ethylene oxide) in the Melt. *Die Makromol. Chemie Macromol. Chem. Phys.* **2334**, 2325–2334 (1983).

14. Nawaz, S. & Carbone, P. Coarse-Graining Poly(ethylene oxide)--Poly(propylene oxide)--Poly(ethylene oxide) (PEO--PPO--PEO) Block Copolymers Using the MARTINI Force Field. *J. Phys. Chem. B* **118**, 1648–1659 (2014).

15. Grunewald, F., Rossi, G., de Vries, A. H., Marrink, S. J. & Monticelli, L. Transferable MARTINI Model of Poly(ethylene Oxide). *J. Phys. Chem. B* **122**, 7436–7449 (2018).

16. Wolf, R. M. & Suter, U. W. Conformational Characteristics of Poly (vinyl alcohol). *Macromolecules* **17**, 669–677 (1984).

17. van Krevelen, D. W. *Properties of Polymers, Correlations with Chemical Structure*. (Elsevier Publication Company, 1972).

18. Ding, Y. & Sokolov, A. P. Comment on the dynamic bead size and Kuhn segment length in polymers: Example of polystyrene. *J. Polym. Sci. Part B Polym. Phys.* **42**, 3505–3511 (2004).

19. Yoon, D. Y., Suter, U. W., Sundararajan, P. R. & Flory, P. J. Conformational Characteristics of Poly(methyl acrylate). *Macromolecules* **8**, 784–789 (1975).

20. Sim, A. Y. L., Lipfert, J., Herschlag, D. & Doniach, S. Salt dependence of the radius of gyration and flexibility of single-stranded DNA in solution probed by small-angle x-ray scattering. *Phys. Rev. E - Stat. Nonlinear, Soft Matter Phys.* **86**, 1–5 (2012).

21. Lindorff-Larsen, K. *et al.* Improved side-chain torsion potentials for the Amber ff99SB protein force field. *Proteins Struct. Funct. Bioinforma.* **78**, 1950–1958 (2010).

22. Abraham, M. J. *et al.* GROMACS: High performance molecular simulations through multi-level parallelism from laptops to supercomputers. *SoftwareX* **1–2**, 19–25 (2015).

23. Properties, B., Nucleic, O. F., At, A., To, R. & Performance, M. Biophysical Properties of Nucleic Acids At Surfaces. **2**, 436–471 (2015).

24. Sethuraman, V., Mogurampelly, S. & Ganesan, V. Ion transport mechanisms in lamellar phases of salt-doped PS–PEO block copolymer electrolytes. *Soft Matter* **13**, 7793–7803 (2017).

25. Bouchet, R. *et al.* Charge transport in nanostructured PS--PEO--PS triblock copolymer electrolytes. *Macromolecules* **47**, 2659–2665 (2014).

26. Yuan, R. *et al.* Ionic conductivity of low molecular weight block copolymer electrolytes. *Macromolecules* **46**, 914–921 (2013).

27. Gekko, K. & Noguchi, H. Physicochemical studies of oligodextran. I. Molecular weight dependence of intrinsic viscosity, partial specific compressibility and hydrated water. *Biopolymers*

10, 1513–1524 (1971).

28. Gasciolli, V., Choplin, L., Paul, F. & Monsan, P. Viscous properties and molecular characterization of enzymatically size-controlled oligodextrans in aqueous solutions. *J. Biotechnol.* **19**, 193–202 (1991).

29. Andes-Koback, M. & Keating, C. D. Complete Budding and Asymmetric Division of Primitive Model Cells To Produce Daughter Vesicles with Different Interior and Membrane Compositions. *J. Am. Chem. Soc.* **133**, 9545–9555 (2011).

30. Basedow, A. M. & Ebert, K. H. Production, characterization, and solution properties of dextran fractions of narrow molecular weight distributions. *J. Polym. Sci. Polym. Symp.* **66**, 101–115 (1979).
