# Supplementary material for: Polyply; a python suite for facilitating simulations of macromolecules and nanomaterials
Source: Nat Commun. 2022 Jan 10;13:68. doi: 10.1038/s41467-021-27627-4 (PMC8748707; doi:10.1038/s41467-021-27627-4)
Supplement: Supplementary file 3 — Reporting Summary [file 41467_2021_27627_MOESM3_ESM.pdf]

nature portfolio

Corresponding author(s): Siewert-Jan Marrink

Last updated by author(s): Nov 16, 2021

Reporting Summary

Nature Portfolio wishes to improve the reproducibility of the work that we publish. This form provides structure for consistency and transparency in reporting. For further information on Nature Portfolio policies, see our Editorial Policies and the Editorial Policy Checklist.

Statistics

For all statistical analyses, confirm that the following items are present in the figure legend, table legend, main text, or Methods section.

n/a	Confirmed	
☐	☒	The exact sample size (*n*) for each experimental group/condition, given as a discrete number and unit of measurement
☐	☒	A statement on whether measurements were taken from distinct samples or whether the same sample was measured repeatedly
☒	☐	The statistical test(s) used AND whether they are one- or two-sided<br>*Only common tests should be described solely by name; describe more complex techniques in the Methods section.*
☒	☐	A description of all covariates tested
☐	☒	A description of any assumptions or corrections, such as tests of normality and adjustment for multiple comparisons
☒	☐	A full description of the statistical parameters including central tendency (e.g. means) or other basic estimates (e.g. regression coefficient) AND variation (e.g. standard deviation) or associated estimates of uncertainty (e.g. confidence intervals)
☒	☐	For null hypothesis testing, the test statistic (e.g. *F*, *t*, *r*) with confidence intervals, effect sizes, degrees of freedom and *P* value noted<br>*Give P values as exact values whenever suitable.*
☒	☐	For Bayesian analysis, information on the choice of priors and Markov chain Monte Carlo settings
☒	☐	For hierarchical and complex designs, identification of the appropriate level for tests and full reporting of outcomes
☒	☐	Estimates of effect sizes (e.g. Cohen's *d*, Pearson's *r*), indicating how they were calculated

Our web collection on statistics for biologists contains articles on many of the points above.

Software and code

Policy information about availability of computer code

Data collection	GROMACS (v. 2019 & 2020), VMD 1.9.4a12, Inkscape 0.54.3, numba >= 0.51.2, networkx=2, scipy>=1.5.4, vermouth >= 0.7.1, matplotlib 3.5, polyply (https://github.com/marrink-lab/polyply_1.0)
Data analysis	Fitting was done using Symfit 0.5.4; additional analysis was done using the gmx polystat part of the GROMACS software (v. 2019, 2020)

For manuscripts utilizing custom algorithms or software that are central to the research but not yet described in published literature, software must be made available to editors and reviewers. We strongly encourage code deposition in a community repository (e.g. GitHub). See the Nature Portfolio guidelines for submitting code & software for further information.

Data

Policy information about availability of data

All manuscripts must include a data availability statement. This statement should provide the following information, where applicable:

- Accession codes, unique identifiers, or web links for publicly available datasets
- A description of any restrictions on data availability
- For clinical datasets or third party data, please ensure that the statement adheres to our policy

The polymer model parameters are part of the polyply library (https://github.com/marrink-lab/polyply_1.0) and all input files and protocols are provided in the tutorial or example folder included with the polyply regression test repository (https://github.com/marrink-lab/polyply_regression_tests). Source data underlying Figure 3 and 4 are provided with this paper.

Field-specific reporting

Please select the one below that is the best fit for your research. If you are not sure, read the appropriate sections before making your selection.

- [x] Life sciences
- [ ] Behavioural & social sciences
- [ ] Ecological, evolutionary & environmental sciences

For a reference copy of the document with all sections, see nature.com/documents/nr-reporting-summary-flat.pdf

Life sciences study design

All studies must disclose on these points even when the disclosure is negative.

Sample size	The number of systems and simulation sampling is enough for this proof of principle study.
Data exclusions	no data was excluded
Replication	Replicas in the context of this study constitute indpendent executions of polyply initialized with a different random state. All replications successfully reproduced the behviour presented in this study.
Randomization	The itp generation is a deterministic program, which has the purpose of exactly reproducing expected results. Thus randomization is not applicable. The structure generation program is itself initated with a different random state, therefore randomizing the outcome, which as shown in the study does not change the expected result.
Blinding	Blinding does not apply since there is no observation bias in the program.

Reporting for specific materials, systems and methods

We require information from authors about some types of materials, experimental systems and methods used in many studies. Here, indicate whether each material, system or method listed is relevant to your study. If you are not sure if a list item applies to your research, read the appropriate section before selecting a response.

Materials & experimental systems

n/a	Involved in the study	
x		Antibodies
x		Eukaryotic cell lines
x		Palaeontology and archaeology
x		Animals and other organisms
x		Human research participants
x		Clinical data
x		Dual use research of concern

Methods

n/a	Involved in the study	
x		ChIP-seq
x		Flow cytometry
x		MRI-based neuroimaging
